# Systemic Symptoms as Potential Predictors of Chronic Neck Pain on Initial Examination: Can Systemic Symptoms Act as a Predictor of Neck Pain?

**DOI:** 10.3390/jpm14070688

**Published:** 2024-06-26

**Authors:** Seo-Hyun Park, Dong-Ho Keum

**Affiliations:** Department of Rehabilitation Medicine of Korean Medicine, Dongguk University Bundang Oriental Hospital, 268, Buljeong-ro, Bundang-gu, Seongnam-si 13601, Gyeonggi-do, Republic of Korea; 20240271@dongguk.ac.kr

**Keywords:** neck pain, predicting factor, upper GI disturbance

## Abstract

Background: Neck pain is a prevalent musculoskeletal disorder that can cause other additional misalignments and other misalignment-induced chronic musculoskeletal diseases. Although numerous risk factors for chronic neck pain have been researched, systemic symptoms have not received the same level of investigation. The aim of this study was to analyze the link between subjective systemic symptoms and neck pain based on initial holistic interviews, with the objective of identifying potential predictive factors for neck pain. Methods: This retrospective cross-sectional study included patients hospitalized due to acute neck pain between January 2018 and August 2021. Data collected included demographic information, treatment details, neck-pain characteristics, medical history, and co-occurring symptoms regardless of their known association with neck pain. Statistical analyses, including independent *t*-tests, Mann–Whitney U tests, chi-squared tests, Fisher’s exact tests, and correlation analyses, were performed. Results: With regard to the demographic characteristics, a significant positive correlation was observed between age and initial pain intensity (*p* < 0.01), while female sex was associated with changes in pain intensity (*p* < 0.05). Past medical conditions, including diabetes, hyperlipidemia, heart attacks, and psychological medical history, demonstrated a significant relationship with neck pain (*p* < 0.01, *p* < 0.05, *p* < 0.05, and *p* < 0.05, respectively). Hospitalization duration, cervical dizziness, limitations in the cervical range of motion (ROM), and widespread pain were significantly associated with neck pain (*p* < 0.05, *p* < 0.05, *p* < 0.01, and *p* < 0.001, respectively). Among the subjective systemic symptoms, only upper gastrointestinal (GI) disturbance displayed a significant association with neck pain (*p* < 0.01). Conclusions: This study identified several potential predictors of neck pain—notably, upper GI disturbances—providing a new avenue to investigate the prognostic factors of neck pain. However, further study is needed to substantiate these findings and elucidate the precise nature of these associations.

## 1. Introduction

Neck pain is typically characterized as discomfort within the neck or shoulder region, often manifesting as an increase in existing pain, heightened sensitivity, a limited range of cervical motion, or as “widespread pain” such as radiating discomfort in the upper extremity [1]. It is well documented that musculoskeletal conditions pose a risk factor for the development of chronic disease. A recent analysis of the Global Burden of Disease (GBD) 2019 data identified neck pain as a significant contributor to the overall burden of musculoskeletal conditions. It has been revealed that neck pain accounts for 2.56% of the total year lived with disability (YLD) [2,3,4,5]. Consistent with this, it is essential to identify the risk factors that can precipitate chronic musculoskeletal conditions and to proactively manage them [2,3,4,5].

Extensive research has been conducted to identify the risk factors for chronic neck pain [6,7,8,9,10,11,12,13,14]. Several studies have suggested that certain neck-pain characteristics such as cervical curvature, the presence of radiating pain, and a limited range of motion (ROM) can elevate the prevalence of chronic neck pain [6]. Additionally, demographic and socio-psychological factors have been implicated as prognostic factors for chronic neck pain. These include an advanced age, female sex, depression, long-term stress, a lack of social support, anxiety-induced psychological stress, sustained posture due to an office environment, and duration [7,8,9,10]. Other studies have also suggested predictive factors that are unrelated to cervical characteristics or socio-psychological elements [11,12]. Sleep disorder has been proposed as a potential risk factor for chronic or severe neck pain, although this relationship remains a point of contention within the field [13,14].

Therefore, we endeavored to identify additional risk factors that could influence the severity or duration of neck pain. In the context of Korean medicine (KM), the foundational theory emphasizes a holistic approach to patient assessments. In KM, there is a concept that the “Decrease of circulation including blood, Qi, and lymph makes pain” [15]. Based on this view, KM doctors try to discover the reason for a decrease in circulation and manage it to reduce the pain. It is common for KM doctors to engage in comprehensive evaluations during their initial consultations with patients, encompassing a broad range of physical and lifestyle aspects [1,15]. This examination extends beyond merely characterizing pain (such as its intensity, distribution, and associated symptoms) and includes inquiries into sleep patterns, dietary habits, gastrointestinal discomforts, bowel regularity, urinary symptoms, and even details like sweating, thirst, and so on [1,15]. Using this comprehensive evaluation, KM doctors endeavor to identify the overarching causes of pain, including the relationship between pain and the region of decreased circulation, to elucidate the pathomechanisms involved and subsequently determine the patients’ “pattern identification (PI)” [1,15].

Dong-Eui-Bo-Kham, the main medical textbook of KM, introduces various causes of low back pain [15,16]. It presents the reasons for low back pain, from acute sprains to excessive food intake [15,16]. There are few studies that have reported the association between low back pain and other gastrointestinal disorders [17]. However, to the best of our knowledge, no studies have focused on the association between neck pain and other systemic symptoms.

This retrospective cross-sectional study was conducted with the primary objective of discerning the predictive factors for neck pain using the comprehensive initial interview approach of KM. More specifically, the aim was to identify the potential factors associated with neck pain following an analysis of patients’ various conditions.

## 2. Methods

### 2.1. Study Design and Participants

This investigation was a retrospective cross-sectional study designed to identify potential prognostic indicators through the analysis of associations between acute neck pain and subjective systemic symptoms.

The study cohort consisted of patients admitted to Dongguk Oriental Hospital in Bundang between January 2018 and August 2021 who presented with acute neck pain. For the purpose of this study, acute neck pain was defined as pain reported for a duration of fewer than three weeks. To extract enough information, including sufficient systemic symptoms from diagnosis, only hospitalized patients were included. Patients were excluded if their hospital stay was fewer than three days or if they did not receive any treatment during their admission (Table 1).

In order to ensure the anonymity of patients and to protect their privacy, a chart review process was undertaken by researchers unrelated to this study researchers. Initially, a researcher extracted the relevant data while omitting all personally identifiable information. Following this, the anonymized data were handed over to another researcher for analysis. This study protocol received approval from the Institutional Bioethics Committees (IRB No. DUBOH 2021-0009).

### 2.2. Data Extraction

The extraction of pertinent information was undertaken from admission and discharge records. This encompassed general information such as age, gender, duration of symptoms, length of hospital stay, smoking history, and the patient’s past medical history. The latter included metabolic conditions (diabetes, hypertension, and hyperlipidemia), history of myocardial infarction, gynecological issues, gastrointestinal disease, respiratory disorders, psychological illnesses, and musculoskeletal disease. The inclusion of these conditions was predicated upon their confirmed diagnosis in the hospital.

Details regarding the pain experienced by patients were also recorded. These characteristics included pain intensity and its fluctuation as well as associated symptoms (headache and dizziness), range of motion (ROM), and the extent of neck pain. Pain intensity was assessed twice, once at admission and once at discharge, using a numeric rating scale (NRS). Variations in pain were calculated in two ways; the first was a simple subtraction denoting the difference in pain levels before and after hospitalization, while the second method involved computing the percentage change in pain intensity from admission to discharge.

Additionally, a systematic review of the patient’s subjective symptoms was carried out using admission records. These findings were based on patient interviews conducted by KM doctors possessing more than one year of clinical experience. Gastrointestinal (GI) disturbances were subdivided into upper and lower GI disturbances. Upper GI disturbances included subjective symptoms complying with the Rome IV Criteria for functional dyspepsia such as epigastric pain, epigastric burning, early satiety, and bothersome post-prandial fullness. Lower GI disturbances encompassed conditions such as constipation and diarrhea. Sleep disturbances, urinary disturbances, and psychological disorders (anxiety or depression) were also documented.

### 2.3. Statistical Analysis

All statistical analyses were conducted using SPSS 12.0 for Windows (SPSS Inc., Chicago, IL, USA). Continuous data were reported as means with their associated standard deviations (SD), while categorical data were presented as frequencies and percentages (*n*%). Where possible, the patients were divided into two groups based on their demographic characteristics (gender and smoking), presence of a medical history, presence of accompanied cervical symptoms, and presence of several subjective systemic symptoms. An independent *t*-test was employed to compare the continuous variables, complemented by a Mann–Whitney U test if the sample size discrepancy between the two groups exceeded a two-fold difference. A chi-squared test or Fisher’s exact test was used, as appropriate, to compare the categorical variables. For a correlation analysis of the continuous variables, Pearson or Spearman’s correlation coefficients were computed. In all cases, a *p*-value less than 0.05 was considered to be statistically significant.

## 3. Results

### 3.1. General Characteristics of Participants

**Figure 1:**
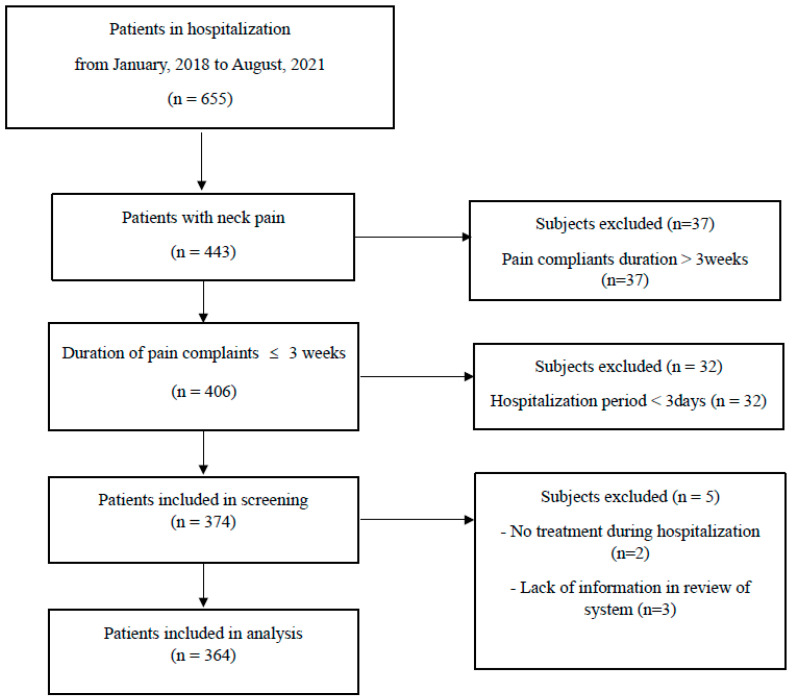
Flow chart.

The study analyzed a total of 364 patients presenting with acute neck pain (Figure 1). The cohort consisted of 223 female and 141 male participants, with an average age of 43.26 ± 0.74 years. The patients reported an average complaint duration of 6.12 ± 2.75 days, while the mean hospitalization period was 8.35 ± 2.06 days. Pain intensity was evaluated twice, at admission and discharge, using the NRS to quantify the change in pain intensity. The initial pain (NRS at admission) was 5.62 ± 0.08 and the pain intensity at discharge (NRS at discharge) was 3.27 ± 0.09. The change in pain intensity (△NRS) was calculated as 2.35 ± 0.09 and the rate of change (△NRS(%)) was determined to be 41.56 ± 1.36% (Table 2).

### 3.2. Association between Demographic Characteristics and Neck Pain

A positive correlation was observed between age and initial pain intensity (*p* < 0.01), although age showed no significant association with the change in pain intensity. Gender had no significant impact on initial pain intensity. However, female patients presented significantly higher pain intensity at discharge (*p* < 0.05). Furthermore, the change in pain intensity and the rate of change in pain intensity were significantly greater in male patients (*p* < 0.05 and *p* < 0.05). Smoking status did not significantly influence pain intensity (Table 3).

### 3.3. Association between Past Medical History and Neck Pain

Patients with diabetes mellitus (DM) or hyperlipidemia exhibited significantly higher initial pain intensity (*p* < 0.01 and *p* < 0.05). Moreover, hyperlipidemic patients displayed a significantly larger change in both the numerical subtraction of pain and the rate of change (*p* < 0.01 and *p* < 0.05). A history of psychological conditions was associated with a smaller change in pain intensity (*p* < 0.05). Participants with a history of myocardial infarction had significantly lower initial pain intensity and a smaller change in pain intensity (*p* < 0.001 and *p* < 0.05). Other past medical conditions such as hypertension (HTN), gynecological disease, gastrointestinal disease, and respiratory disease demonstrated no significant relationship with neck pain. Additionally, a past history of musculoskeletal disease, including conditions affecting the neck or other spinal regions, did not result in significant differences in pain intensity (Table 4).

### 3.4. Association between Neck-Pain Characteristics and Pain Intensity

The analysis indicated a negative correlation between the duration of neck-pain symptoms and both pain intensity and changes in pain. However, these relationships were not statistically significant. The duration of treatment, defined here as the period of hospitalization, demonstrated a positive correlation between initial pain intensity and changes in pain, including both absolute and relative variations (*p* < 0.01, *p* < 0.01, and *p* < 0.05, respectively) (Table 5a).

Symptoms like headaches or dizziness, which are likely to originate from the cervical region, were found to have no statistically significant associations with pain intensity. Nevertheless, patients experiencing dizziness exhibited a notably lower percentage in pain reduction (*p* < 0.05). Cervical ROM limitations, indicative of a neck disorder, were strongly associated with neck pain. Patients with a limited cervical ROM reported significantly greater initial pain intensity (*p* < 0.001) and a more pronounced change in pain (*p* < 0.01). Furthermore, the spatial distribution of pain—whether localized in the cervical region or widespread, including the mid-back and arm regions—showed a significant association with neck pain. Patients with a widespread distribution of pain exhibited higher pain intensity levels both at admission and discharge (*p* < 0.001 and *p* < 0.001, respectively) and a smaller relative change in pain (*p* < 0.05) (Table 5b).

### 3.5. Association between Subjective Systematic Symptoms and Neck Pain

Significant associations were observed between upper GI disturbances and elevated neck-pain intensity upon both admission and discharge (*p* < 0.01 and *p* < 0.001, respectively). Patients with these disturbances also exhibited a diminished rate of change in their pain intensity (*p* < 0.01). However, the presence of lower GI disturbances such as constipation or diarrhea did not yield any significant associations with neck-pain intensity. Similarly, no significant correlations were identified between neck pain and other subjective symptoms, encompassing sleep and urinary disturbances as well as psychological symptoms like anxiety and depression (Table 6).

### 3.6. Correlation between Subjective Symptoms and Neck-Pain Characteristics

Subjective symptoms associated with upper GI disturbances displayed significant correlations with certain characteristics of neck pain. Specifically, higher incidences of cervical dizziness, a limited ROM, and distributed neck pain were reported among patients presenting upper GI disturbances (*p* < 0.001, *p* < 0.05, and *p* < 0.01, respectively). Regarding the distribution of neck pain, a strong correlation was observed with systemic symptoms. More precisely, the presence of sleep disturbance, upper GI disturbance, lower GI disturbance, and constipation were associated with diffused neck pain (*p* < 0.05, *p* < 0.001, *p* < 0.05, and *p* < 0.05, respectively) (Table 7).

## 4. Discussion

In this study, we found that the characteristics of neck pain, including initial pain intensity, a restricted ROM, and distribution of pain, could affect the prognosis of neck pain. Furthermore, we also found that other systemic symptoms, especially upper GI disturbances, could also affect the prognosis of neck pain. Numerous efforts have been undertaken to identify potential predictive factors for the prognosis of neck pain; however, this is the first study to focus on systemic symptoms, including gastrointestinal disturbances or other symptoms.

Our study found that, in addition to demographic attributes, the female sex was associated with increased pain intensity at the time of discharge and a less significant decrease in pain during hospitalization. This result, consistent with prior research [7], was evaluated using two measures: the difference in the numeric scale and the percentage. Conversely, age significantly affected only initial pain intensity, whereas smoking demonstrated no association with pain intensity at any time point or in terms of changes in pain intensity. This lack of a long-term relationship might stem from our research design, which concentrated on initial pain intensity and changes following short-term treatment.

Upon investigating medical histories, conditions such as DM, hyperlipidemia, and previous myocardial infarctions were found to be associated with a higher initial pain intensity. Interestingly, hyperlipidemia and prior myocardial infarctions also demonstrated a significant relationship with changes in pain intensity. These conditions are associated with circulatory issues and have been reported to potentially induce micro-inflammation [18,19,20], which could explain a slower decrease in pain. This study, however, contradicted previous reports regarding the lack of significant differences in pain intensity among patients with psychological histories. Several factors might account for this discrepancy. Firstly, most patients in our study with psychological histories were receiving treatment and thus their symptoms were well managed; secondly, our focus on short-term changes and the acute phase might have under-represented the negative impacts of these histories on pain intensity; and finally, potential statistical errors arising from a significant disparity in sample sizes between the groups and the lack of a normal distribution could have influenced the results.

In examining the characteristics of pain, our findings largely aligned with previous studies. Both the duration of hospitalization and initial pain intensity were positively correlated with initial pain intensity and changes in pain intensity. Other related symptoms such as a limited ROM and widespread neck pain were associated with higher initial pain intensity and larger fluctuations in pain intensity. Based on previous research, the significant difference in initial pain can be understood. The greater changes in pain intensity among patients with ROM limitations might have been a result of their higher initial pain intensity. Conversely, smaller changes in pain intensity among those with widespread pain could indicate a need for more active treatment interventions. Notably, the pain intensity for patients with cervical pain associated with cervical headaches and dizziness was higher than that of the control group, although these differences did not reach a statistical significance.

Our study found a noteworthy link between upper GI disturbances and pain intensity both pre- and post-treatment as well as changes in the percentages of pain intensity. Contrary to previous studies, no significant associations were found between pain intensity and sleep disturbance, psychological symptoms, lower GI disturbances, or urinary disturbances. A more detailed analysis investigating the relationship between systematic symptoms and neck-pain characteristics revealed that upper GI disturbances also exhibited significant correlations with widespread pain.

Several explanations may be proposed to elucidate the association between upper GI disturbances and neck pain. The first concerns the autonomic nervous system and its response to stress. In this study, our focus was on subjective symptoms, which, in the context of upper GI disturbances, included post-prandial fullness, early satiation, gastric pain, epigastric burning, and other forms of upper abdominal discomfort, as identified using patient interviews. Although this study gathered subjective symptoms and did not strictly diagnose functional dyspepsia based on the Rome IV Criteria, it is well established that functional dyspepsia correlates with the functioning of autonomic nerves [21,22,23]. Previous research has also suggested a relationship between the functionality of the autonomic nervous system and the mechanical strain of the musculoskeletal system [24].

Previous studies investigating the autonomic nervous system and pain have reported a negative correlation between neck-pain intensity and the activation of the sympathetic nervous system. Conversely, they have identified a positive relationship between pain intensity and the activation of the parasympathetic nervous system [24,25]. Furthermore, studies exploring the association between neck pain and stress have consistently reported increased levels of stress among patients suffering from neck pain [26,27,28]. Although this study did not find a significant correlation between acute stress symptoms and the intensity of neck pain, it is widely accepted that stress is a prominent risk factor for neck pain.

The sympathetic ganglion comprises the paravertebral ganglion, prevertebral ganglion located in the abdominal cavity, pelvic region, and anterior to the vertebra and the terminal ganglion [29,30]. The spinal nerve emanates from the spinal cord, extending to the peripheral region via the intervertebral foramen [29,30]. When passing through the intervertebral foramen, the spinal nerve can be subjected to compression by paravertebral structures or traction can occur along its route to the peripheral region due to various causes [29,30]. These external stimuli to the spinal nerve can trigger a response in the segment of the autonomic nerve located in the paravertebral region, potentially leading to a subsequent depression of the autonomic nervous system [29,30]. The malfunction of the autonomic nervous system can be a consequence of mechanical problems associated with musculoskeletal disorders. Conversely, paravertebral compression can also arise from somatic problems, including conditions such as functional dyspepsia. The regulation of the autonomic system is managed by feedback from the sympathetic and parasympathetic nerves, alongside signals from internal and sensory organs. If this feedback triggers the sympathetic ganglion in the paravertebral region, it may result in the hyperactivation of the sympathetic nerve. This can lead to persistent musculoskeletal issues, pain, and disorders. Given that pain can be initiated and perpetuated by mechanical musculoskeletal problems, afferent signals from peripheral receptors, and the central nervous system, continuous harmful stimulation may result in central sensitization [29,30].

Another possible factor is the segmental association between the upper GI tract and the neck. The phrenic nerve, a combination of motor and sensory fibers [29,30], originates from the C3–C5 spinal nerves in the neck and innervates the upper GI tract [29,30]. Considering that feedback mechanisms can occur bidirectionally—on both afferent and efferent sides—this regional association is noteworthy [29,30]. Dysfunction in the upper GI tract can activate the sympathetic nerve ganglion in the lower thoracic region, sending a stimulating signal to upper segmental levels [31,32]. Once the afferent signal is activated in the upper thoracic region—specifically, T1 to T5—it advances to the cervical region, forming a synapse on the verve ganglion there [29,30].

The final potential explanation may be attributable to muscle strain. It must be noted that certain cervical muscles such as the trapezius and sternocleidomastoid (SCM) as well as masticatory muscles, scalene, and the cervical and thoracic erectus muscles can be readily activated by shifts in the autonomic nervous system [33]. The hyperactivation of these muscles can induce and sustain myofascial pain in the neck region [15,33]. Additionally, these muscles are implicated in abnormal breathing patterns. Thoracic cage and diaphragm movements during respiration are correlated with pain and misalignment [34,35]. Upper GI symptoms, on the other hand, are associated with diaphragm dysfunction, which can lead to abnormalities in thoracic respiration, including thorax elevation [36,37,38,39]. If muscles such as the SCM, upper trapezius, scalene, serratus anterior, and pectoralis—which are known to have associations with rounded shoulder or upper crossed syndrome—are hyperactivated, this can result in a painful situation accompanied by misalignment [40].

This study presents several limitations that should be acknowledged. The causal relationship between neck pain and other subjective symptoms is unknown. We found that there was an association between neck pain and some subjective systemic symptoms; however, which one preceded is controversial. Secondly, our focus was on acute symptoms presenting within three months of the onset of pain and assessments were conducted only once, at the initial interview. Hence, our findings proposed predictive factors based on short-term effects. Future research with a more extended follow-up period is required to investigate the potential long-term associations. We analyzed subjective symptoms based on patient interviews and did not classify groups according to patients’ histories of other diseases. This resulted in a lack of objective data and some outcome measurements did not follow a normal distribution. Therefore, further well-designed studies with stringent inclusion criteria, repeat assessments of objective symptoms, and larger sample sizes are necessary.

Despite these limitations, our study possesses several noteworthy attributes. Foremost, this is the first initiative to identify novel predictive factors for neck pain using subjective systemic symptoms. To the best of our knowledge, no previous study has reported a potential association between neck pain and upper GI dysfunction.

Emphasizing initial systemic symptoms and identifying risk factors for severe or persistent pain at the time of first diagnosis could be a groundbreaking approach to developing proactive treatments and preventing the progression to chronic pain.

In conclusion, subjective systemic symptoms, including upper GI symptoms, may serve as potential predictive factors for chronic and severe neck pain. Although future studies are required to elucidate the association between upper GI symptoms and neck pain, it is certainly meritorious to place an increased emphasis on systemic symptoms at the initial diagnosis.

## 5. Conclusions

This study analyzed the relationship between subjective systemic symptoms and changes in neck pain. We found several possible factors that could affect the prognosis of neck pain. The first was the characteristics of neck pain such as initial pain intensity, a limited ROM, and distribution of neck pain. This was in alignment with previous studies that suggested the characteristics of neck pain are a predictive factor for chronic neck pain. Another interesting finding was upper GI disturbance. We found a relationship between the presence of upper GI disturbances and prolonged neck pain. Although we do not know which one was first, it is noteworthy that subjective systemic symptoms, including an upper GI disturbance, can be a possible predictive factor of prolonged neck pain. It indicates the importance of personalized deep examinations, focusing on subjective systemic symptoms. Although further well-designed studies are needed, it can be proposed that subjective systemic symptoms, including upper GI symptoms, may serve as potential predictive factors for chronic and severe neck pain.

## Figures and Tables

**Table 1:** Inclusion and exclusion criteria of study.

Inclusion Criteria	Exclusion Criteria
- Duration of pain complaints ≤ 3 weeks - Hospitalization period ≥ 3 days	- Pain complaint duration > 3 weeks - Hospitalization period < 3 days - No treatment during hospitalization - Lack of information in review of system

**Table 2:** General characteristics of subjects.

N	364
Age (year)	46.26 ± 0.74
Gender (female/male)	223/141 (61.3/38.7)
Pain complaint duration (day)	6.12 ± 2.75
Hospitalization period (day)	8.35 ± 2.06
NRS at admission	5.62 ± 0.08
NRS at discharge	3.27 ± 0.09
△NRS	2.35 ± 0.09
△NRS(%)	41.56 ± 1.36

Data presented as mean ± standard deviation or number (%). △NRS: NRS at admission − NRS at discharge. △NRS(%): [(NRS at admission − NRS at discharge)/NRS at admission].

**Table 3:** The relationship between demographic characteristics and neck pain.

		NRS at Admission	NRS at Discharge	△NRS	△NRS(%)
Gender	Male (141)	5.59 ± 1.58	2.99 ± 1.69	2.60 ± 1.76	45.68 ± 27.43
	Female (223)	5.64 ± 1.54	3.45 ± 1.68	2.20 ± 1.64	38.96 ± 24.68
	*p*-Value	0.753	0.011 *	0.033 *	0.016 *
Age	Age †	0.148	0.042	0.096	0.044
	*p*-Value	0.005 **	0.423	0.068	0.407
Smoking	Smoking (41)	5.85 ± 1.65	3.54 ± 1.69	2.32 ± 1.62	38.86 ± 25.71
	Non-smoking (323)	5.59 ± 1.54	3.24 ± 1.70	2.38 ± 1.71	41.91 ± 26.00
	*p*-Value	0.308	0.294	0.881	0.479

Data presented as mean ± standard deviation. *p*-Value for gender calculated using independent *t*-test (* = *p* < 0.05). *p*-Value for smoking evaluated using Mann–Whitney U test. †: correlation coefficient calculated using a correlation analysis (** = *p* < 0.01). △NRS: NRS at admission − NRS at discharge. △NRS(%): [(NRS at admission − NRS at discharge)/NRS at admission].

**Table 4:** The relationship between medical history and neck pain.

		NRS at Admission	NRS at Discharge	△NRS	△NRS(%)
DM	DM (16)	6.63 ± 1.71	3.31 ± 1.85	3.31 ± 1.12	48.42 ± 26.51
	Control (348)	5.57 ± 1.53	3.27 ± 1.69	2.31 ± 1.66	41.25 ± 25.92
	*p*-Value	0.008 **	0.927	0.081	0.280
HTN	HTN (57)	5.86 ± 1.74	3.44 ± 2.00	2.42 ± 1.73	41.42 ± 26.47
	Control (307)	5.58 ± 1.51	3.24 ± 1.63	2.34 ± 1.69	41.59 ± 25.89
	*p*-Value	0.206	0.491	0.747	0.965
Hyperlipidemia	Hyperlipidemia (43)	6.16 ± 1.63	3.12 ± 1.75	3.05 ± 1.76	49.98 ± 23.76
	Control (321)	5.55 ± 1.53	3.30 ± 1.69	2.26 ± 1.67	40.57 ± 26.10
	*p*-Value	0.015 *	0.515	0.004 **	0.046 *
Heart attack	Heart attack (25)	4.56 ± 1.71	3.08 ± 1.55	1.64 ± 2.14	35.28 ± 24.73
	Control (339)	5.70 ± 1.51	3.29 ± 1.71	2.41 ± 1.65	42.03 ± 26.01
	*p*-Value	0.000 ***	0.552	0.029 *	0.210
Psychological history	Psychological history (23)	5.35 ± 1.72	3.70 ± 2.12	1.65 ± 2.41	34.49 ± 30.90
	Control (341)	5.64 ± 1.54	3.25 ± 1.66	2.40 ± 1.63	42.04 ± 25.56
	*p*-Value	0.384	0.219	0.040 *	0.177
Gynecological disease	Gynecological disease (33)	5.45 ± 1.66	3.27 ± 1.57	2.18 ± 1.16	40.08 ± 20.71
	Control (331)	5.64 ± 1.54	3.27 ± 1.71	2.37 ± 1.74	41.71 ± 26.43
	*p*-Value	0.519	0.994	0.399	0.676
Gastrointestinal disease	Gastrointestinal disease (42)	5.43 ± 1.29	3.12 ± 1.73	2.31 ± 1.37	44.05 ± 24.04
	Control (322)	5.65 ± 1.58	3.30 ± 1.69	2.36 ± 1.73	41.24 ± 26.20
	*p*-Value	0.394	0.527	0.828	0.510
Respiratory disease	Respiratory disease (8)	5.50 ± 1.41	3.13 ± 0.64	2.38 ± 1.30	39.79 ± 19.90
	Control (356)	5.62 ± 1.56	3.28 ± 1.71	2.35 ± 1.70	41.60 ± 26.09
	*p*-Value	0.824	0.545	0.972	0.845
Musculoskeletal disease—cervical region	Musculoskeletal disease (35)	5.94 ± 1.33	3.63 ± 1.26	2.31 ± 1.55	36.86 ± 21.36
	Control (329)	5.59 ± 1.57	3.24 ± 1.73	2.36 ± 1.71	42.06 ± 26.37
	*p*-Value	0.197	0.194	0.883	0.260
Musculoskeletal disease—thoracic or lumbar spinal region	Musculoskeletal disease (75)	5.81 ± 1.55	3.47 ± 1.66	2.35 ± 1.56	40.26 ± 24.50
	Control (289)	5.57 ± 1.55	3.22 ± 1.70	2.36 ± 1.73	41.90 ± 26.34
	*p*-Value	0.228	0.272	0.965	0.626
Musculoskeletal disease—non-spinal region	Musculoskeletal disease (52)	5.83 ± 1.42	3.10 ± 1.71	2.73 ± 1.54	47.66 ± 23.17
	Control (312)	5.59 ± 1.57	3.30 ± 1.69	2.29 ± 1.71	40.55 ± 26.28
	*p*-Value	0.302	0.545	0.972	0.845

Data presented as mean ± standard deviation. *p*-Values evaluated using Mann–Whitney U test (* = *p* < 0.05, ** = *p* < 0.01, and *** = *p* < 0.001). △NRS: NRS at admission − NRS at discharge. △NRS(%): [(NRS at admission − NRS at discharge)/NRS at admission].

**Table 5:** (**a**) The relationship between characteristics of pain and change in pain. (**b**) The relationship between pain intensity and accompanied cervical symptoms.

**(a)**					
		**NRS at Admission**	**NRS at Discharge**	**△** **NRS**	**△** **NRS(%)**
Duration of pain complaint		−0.011 (0.829)	0.038 (0.470)	−0.049 (0.351)	−0.054 (0.301)
Duration of hospitalization		0.147 (0.005) **	−0.011 (0.832)	0.159 (0.002) **	0.118 (0.024) *
Initial pain intensity		-	0.467 (<0.01) **	0.451 (<0.01) **	0.078 (0.138)
**(b)**					
		**NRS** **at Admission**	**NRS** **at Discharge**	**△** **NRS**	**△** **NRS(%)**
Headache	Headache (175)	5.61 ± 1.47	3.24 ± 1.55	2.39 ± 1.60	41.49 ± 24.77
	Control (189)	5.63 ± 1.62	3.31 ± 1.82	2.32 ± 1.78	41.63 ± 27.05
	*p*-Value	0.911	0.706	0.712	0.958
Dizziness	Dizziness (128)	5.59 ± 1.50	3.44 ± 1.57	2.17 ± 1.84	37.74 ± 26.92
	Control (236)	5.64 ± 1.58	3.19 ± 1.76	2.45 ± 1.60	43.64 ± 25.22
	*p*-Value	0.752	0.163	0.130	0.038 *
ROM limitation	ROM limitation (192)	5.93 ± 1.54	3.35 ± 1.70	2.60 ± 1.80	43.42 ± 25.15
	Control (172)	5.28 ± 1.50	3.19 ± 1.69	2.08 ± 1.52	39.49 ± 26.73
	*p*-Value	0.000 ***	0.378	0.003 **	0.149
Distribution of neck pain	Widespread (163)	5.95 ± 1.49	3.63 ± 1.53	2.32 ± 1.53	38.03 ± 22.57
	Localized (201)	5.35 ± 1.56	2.99 ± 1.77	2.38 ± 1.82	44.42 ± 28.12
	*p*-Value	0.000 ***	0.000 ***	0.720	0.019 *

(a) Data presented as correlation coefficient (*p*-value). *p*-Values calculated using a correlation analysis (* = *p* < 0.05 and ** = *p* < 0.01). △NRS: NRS at admission − NRS at discharge. △NRS(%): [(NRS at admission − NRS at discharge)/NRS at admission]. (b) Data presented as mean ± standard deviation. *p*-Values evaluated using an independent *t*-test (* = *p* < 0.05, ** = *p* < 0.01, and *** = *p* < 0.001). △NRS: NRS at admission − NRS at discharge. △NRS(%): [(NRS at admission − NRS at discharge)/NRS at admission].

**Table 6:** The relationship between systematic subjective symptoms and neck pain.

		NRS at Admission	NRS at Discharge	△NRS	△NRS(%)
Upper GI disturbance	Upper GI disturbance (168)	5.82 ± 1.47	3.63 ± 1.67	2.18 ± 1.68	36.64 ± 25.51
	Control (196)	5.45 ± 1.61	2.97 ± 1.66	2.50 ± 1.70	45.75 ± 25.63
	*p*-Value	0.027 *	0.000 ***	0.076	0.001 **
Lower GI disturbance	Lower GI disturbance (72)	5.78 ± 1.53	3.56 ± 1.82	2.22 ± 1.57	38.95 ± 25.65
	Control (292)	5.58 ± 1.56	3.21 ± 1.66	2.39 ± 1.72	42.21 ± 26.02
	*p*-Value †	0.339	0.117	0.460	0.342
Sleep disturbance	Sleep disturbance (245)	5.64 ± 1.55	3.31 ± 1.70	2.34 ± 1.70	41.43 ± 25.95
	Control (119)	5.59 ± 1.57	3.20 ± 1.68	2.39 ± 1.69	41.92 ± 26.06
	*p*-Value	0.780	0.567	0.801	0.853
Urinary disturbance	Urinary disturbance (86)	5.56 ± 1.52	3.50 ± 1.69	2.06 ± 1.83	36.84 ± 26.34
	Control (278)	5.64 ± 1.56	3.21 ± 1.69	2.45 ± 1.64	43.02 ± 25.73
	*p*-Value †	0.668	0.159	0.063	0.053
Psychological symptoms	Psychological disturbance (45)	5.33 ± 1.65	3.20 ± 1.62	2.13 ± 1.63	38.66 ± 25.69
	Control (319)	5.66 ± 1.54	3.29 ± 1.71	2.39 ± 1.70	41.97 ± 26.00
	*p*-Value †	0.185	0.753	0.350	0.423

Data presented as mean ± standard deviation. *p*-Values evaluated using an independent *t*-test (* = *p* < 0.05, ** = *p* < 0.01, and *** = *p* < 0.001). †: *p*-Values evaluated using a Mann–Whitney U test. △NRS: NRS at admission − NRS at discharge. △NRS(%): [(NRS at admission − NRS at discharge)/NRS at admission].

**Table 7:** Correlations between subjective symptoms and characteristics of neck pain.

		Headache		Dizziness		ROM Limitation		Widespread Pain	
		N/Y (%)	*p*-Value	N/Y (%)	*p*-Value	N/Y (%)	*p*-Value	N/Y (%)	*p*-Value
Upper GI disturbance	N	110/79 (58.2/41.8)	0.084	144/92 (61.0/39.0)	0.000 ***	104/68 (60.5/39.5)	0.016 *	121/80 (60.2/39.8)	0.007 **
	Y	83/89 (49.1/50.9)		52/76 (40.6/59.4)		92/100 (47.9/52.1)		75/88 (46.0/54.0)	
Lower GI disturbance	N	150/39 (79.4/20.6)	0.672	187/49 (79.2/20.8)	0.524	142/30 (82.6/17.4)	0.290	169/32 (84.1/15.9)	0.040 *
	Y	142/33 (81.1/18.9)		105/23 (82.0/18.0)		150/42 (78.1/21.9)		123/40 (75.5/24.5)	
Urinary disturbance	N	139/50 (73.5/26.5)	0.188	185/51 (78.4/21.6)	0.220	132/40 (76.7/23.3)	0.875	152/49 (75.6/24.4)	0.709
	Y	139/36 (79.4/20.6)		93/35 (72.7/27.3)		146.46 (76.0/24.0)		126/37 (77.3/22.7)	
Sleep disturbance	N	623/126 (33.3/66.7)	0.787	85/151 (36.0/64.0)	0.067	62/110 (36.0/64.0)	0.198	77/124 (38.3/61.7)	0.011 *
	Y	56/119 (32.0/68.0)		34.94 (26.6/73.4)		57/135 (29.7/70.3)		42/121 (25.8/74.2)	
Psychological symptoms	N	170/149 (53.3/46.7)	0.202	212/107 (66.5/33.5)	0.096	151/168 (47.3/52.7)	0.933	176/143 (55.2/44.8)	0.961
	Y	19/26 (42.2/57.8)		24/21 (53.3/46.7)		21/24 (46.7/53.3)		25/20 (55.6/44.4)	

Data presented as n (%). *p*-Values calculated using a chi-squared test (* = *p* < 0.05, ** = *p* < 0.01, and *** = *p* < 0.001).

## Data Availability

Data are not available due to ethical reasons.

## Abbreviations

NRS: numeric rating scale; ROM: range of motion; GI: gastrointestinal; KM: Korean medicine.
